# Process evaluation of TeamUp: a movement-based psychosocial intervention for refugee children in the Netherlands

**DOI:** 10.1186/s13033-021-00450-6

**Published:** 2021-03-19

**Authors:** Alexandra C. E. Bleile, Gabriela V. Koppenol-Gonzalez, Katia Verreault, Karin Abeling, Elin Hofman, Willem Vriend, Adnan Hasan, Mark J. D. Jordans

**Affiliations:** 1 War Child Holland, Amsterdam, The Netherlands; 2 Amsterdam Institute of Social Science Research, University of Amsterdam, Amsterdam, The Netherlands

**Keywords:** Psychosocial support, Movement-based activities, Movement, Children, Refugees, Conflict-affected, Well-being, Resilience

## Abstract

**Background:**

Nearly 60,000 people applied for asylum in the Netherland in 2015, confronting the governmental structures and services with great administrative, logistical and service provision challenges. Refugee children’s psychosocial needs and wellbeing are often overlooked, and post-migration support is of pivotal importance.

**Methods:**

An easy accessible movement–based psychosocial intervention, called TeamUp, was developed for children aged 6–17 living in refugee reception centres. A mixed-method process evaluation was conducted of (1) implementation process, assessing attendance (n = 2183 children, and n = 209 children); (2) implementation quality, using structured observations at two time points to evaluate facilitator’s (2a) individual-level fidelity (n = 81 facilitators); (2b) team-level fidelity (n = 22 teams); (2c) facilitators’ competencies (n = 81); (2d) trainee perceived self-efficacy pre-post training (n = 73); and (3) perceptions on implementation and outcomes, employing a survey (n = 99), focus group discussions and key informant interviews with children (n = 94), facilitators (n = 24) and reception centre staff (n = 10).

**Results:**

Attendance lists showed a mean of 8.5 children per session, and children attending 31.3% of sessions. Structured observations demonstrated 49.2% and 58.2% individual-level fidelity, 72.5% and 73.0% team-level fidelity, and 82.9% and 88.4% adequacy in competencies, each at T1 and T2 respectively. The main reported challenges included managing children’s energy regulation (e.g. offering settling moments) and challenging behaviour. Training participation significantly improved perceived self-efficacy for trainees. The facilitator survey demonstrated on average, high satisfaction and self-efficacy, low experienced burden, and high perceived capacity-building support. Qualitatively, TeamUp was positively perceived by all stakeholders and was regarded as contributing to children’s psychosocial outcomes.

**Conclusion:**

(1) Attendance and group size were lower than expected. (2) The intervention’s facilitator fidelity ranged from moderate to adequate—exhibiting a need for specific fidelity and capacity strengthening—while facilitator competencies were high. Trainee’s perceived self-efficacy improved significantly following a 2-day training. (3) Facilitators expressed high levels of satisfaction, self-efficacy and support, and low burden. The intervention was positively perceived by all stakeholders and to have a positive impact on children’s psychosocial learning and wellbeing.

**Supplementary Information:**

The online version contains supplementary material available at 10.1186/s13033-021-00450-6.

## Introduction

In 2015, the number of refugees arriving in Europe increased drastically. In the Netherlands alone, nearly 60,000 people applied for asylum [28], among which almost 19,000 children [11], confronting the Dutch government with multifaceted challenges [28].

Children on the move, including unaccompanied minors, are particularly vulnerable during the different phases of migration [9, 17]. Even when basic services (shelter, food, education) are provided, children’s and adolescents’ psychosocial needs are often left unidentified and unaddressed, potentially leading to long-term consequences for their wellbeing [58]. Despite arriving in a stable and safe country like the Netherlands, the complexity of the asylum system contributes to continued uncertainty about the future, further affecting people’s mental health when needs are overlooked [21, 37]. The prevalence of emotional and behavioural disorders among refugee children arriving in Europe, ranges from 19.8 to 35.0%. [33]. The accumulation of multiple risk factors and particularly experienced, witnessed or feared violence, may lead to worse health outcomes [10, 19, 57]. Stressors as well as adversity experienced prior and during migration have a substantial effect on children’s wellbeing. Providing safety and support *post*-migration is important to prevent longer term consequences [18, 55]. International policy [29, 62] and multidisciplinary research have recommended, guided and shaped the implementation of mental health and psychosocial support (MHPSS) interventions for over a decade [65]. Offering adequate services to a multilingual, and multicultural population on the move remains challenging, yet all the more important to be part of humanitarian support—particularly for children [29, 62, 65].

Rigorous intervention studies and implementation evaluations of psychosocial support programmes are crucial to strengthen the connection of research and practice as well as to further build evidence base for MHPSS programming [22, 25, 30, 31, 40, 53, 63–65], including movement-based interventions [3, 4, 45]. Research suggests that movement-based activities may facilitate the release of stress and tension in the body [15, 20, 70] as well as reconnecting children to themselves to their peers in non-verbal ways [3, 20, 49]. Further, these non-verbal activities can stimulate neuroplasticity and strengthen existing physiological and psychological resources to restore a sense of wellbeing [15, 20, 49]. Moreover, non-verbal modalities are transcultural, thereby accessible and applicable to all cultures and contexts [20].

To respond to the psychosocial needs of children on the move and address a gap in available services, an easy-accessible movement–based psychosocial intervention, called TeamUp, was developed. The intervention intended to offer structure, stability, normalcy, and socialisation opportunities by means of structured sessions of movement-based activities, which target 6-17 year old children living in refugee reception centres. This study aimed to evaluate the implementation of the TeamUp intervention for refugee children, in order to inform future development, evaluation and scaling.

## Methods

### Setting

The study was conducted in 15 asylum seekers centres in the Netherlands. The selection included a variety of geographical regions (north, south, east, west), settings (city, rural) and types of reception centres. Data were collected between September 2018 and February 2019, attendance lists were included from September 2018 to July 2019.

### Study design

Following the first phases of implementation and monitoring (between 2016 and 2018), a process evaluation was conducted, with the aim to explore how the intervention is delivered, examining implementation fidelity, its challenges and how it can be improved [12]; cf. [46, 60]. The current mixed-methods process evaluation focused on three dimensions, employing multiple methods and using various samples (see Table 1): (1) evaluation of the implementation process: attendance (2) evaluation of the implementation quality: facilitator fidelity [7], competencies [13, 14, 32, 34, 35] and self-efficacy, and (3) evaluation of perceived implementation and outcomes: stakeholders’ overall perceptions of the implementation and perceived outcomes of the intervention. Quantitative pre-post data collection on child level was not possible due to the intervention’s open group approach and frequent relocation of the refugee population.

**Table 1:**
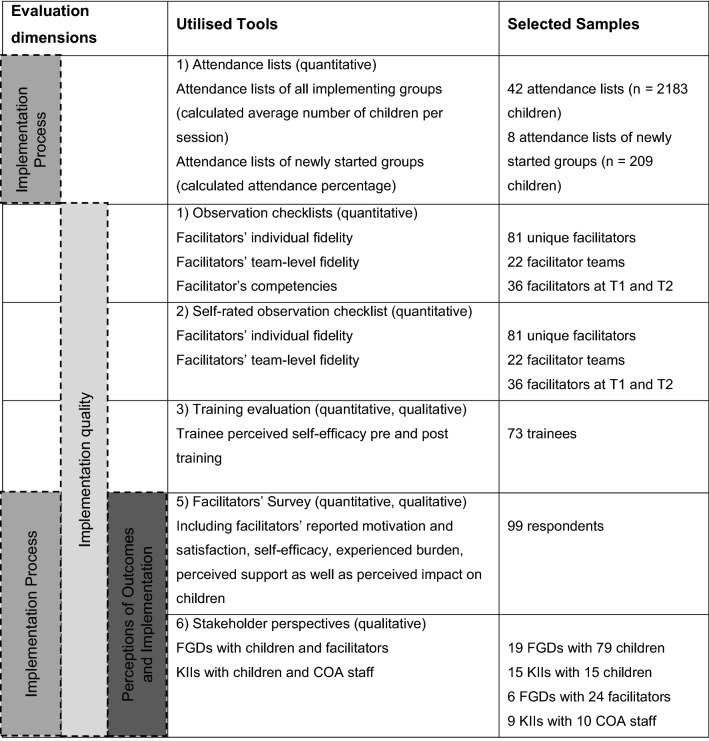
Evaluation Dimensions, Utilised Tools and Selected Samples

FGD: focus group discussion; KII: key informant interview; COA: Dutch abbreviation for ‘Centraal Orgaan Opvang Asielzoekers’, i.e. the Dutch ‘Central Agency for the Reception of Asylum Seekers’

### TeamUp intervention

TeamUp aims to offer children safety, normalcy and structure. Facilitators provide children the space to release stress and tension in their bodies while offering opportunities for social interaction and resource-building, ultimately striving to promote wellbeing and resilience [39, 41, 42]. TeamUp’s theoretical and conceptual underpinnings include guidelines for Mental Health and Psychosocial Support (MHPSS) in emergency settings [29], trauma-informed care principles [27], and the value of the body, movement and play to support socialisation, attunement and self-regulation abilities [51, 69].

The intervention’s methodology consists of a wide variety of group movement-based games, sports-based activities, creative movement and body awareness practices. The sessions follow a guiding structure—(1) opening routine and check-in, (2) a body warm-up, (3) main activities (4) a cooling-down (5) a check-out and closing routine [16], and facilitators use four basic facilitation techniques—flow, build-up, group organisation and demonstration. The non-verbal modality (little use of language) of TeamUp allows inclusivity of children from diverse backgrounds and with different abilities to participate—particularly important for multi-cultural settings [23]. Creating a sense of connectedness, safety and self-efficacy while developing self-soothing and regulating abilities is key in establishing trauma-informed care with vulnerable populations [27, 41]. Specifically, the focus on the body-mind interrelatedness integrates a neurophysiological perspective: through playing and moving, children engage their nervous system by mobilising their bodies. This allows them to shift from possible fight-flight-freeze responses in their bodies (i.e. push, run, hide) to reconnect to calmer states and social engagement mechanisms (e.g. attunement), without the need for verbal processing [38, 52, 69]. Furthermore, the non-judgemental approach creates a safe ‘holding’ space [70] where children are invited to participate, try new activities and ways of being in their bodies. The interaction with adults who model care and self-regulation is crucial for creating and maintaining this safe space [1, 2, 41]. Lastly, the intervention can be contextually and culturally adapted for various populations, particularly important for resilience-focused interventions and settings where services are scarce [59, 66].

Sessions are based on eight psychosocial themes that are addressed through specific behaviour and observable skills (i.e. fear, anger, respect, conflict, bullying, friendship, stress & tension, and assertiveness). They offer different playful, activating games, in different group formations (e.g. individual, pair, small group or full group activities) combined with more calming activities such as mirroring and settling routines [6, 50, 67]. The choice of different activities in the sessions offer opportunities for connectedness, attunement, synchrony, rhythmicity and grounding [36, 43, 70]. Further, children move between various “feel-able”, observable states in the body (i.e. recognising sensations of anger, sadness, joy, fear) through activation, mobilisation, settling and self-expression activities [8, 38, 44, 54]. The weekly sessions are offered by a team of 3–5 trained facilitators for children of a specific age group, e.g. 6–9, 10–14 or 15–17 year olds.

Facilitators are selected volunteers from the proximity of the asylum seeker centre and (a) are minimum 18 years of age, (b) have relevant training, e.g. in psychology, child studies, education, sports, games and/or dance; (c) hold prior experience in facilitating activities with children; (d) show strong interpersonal, intercultural and communication skills, and (e) are requested to commit to the programme for a minimum of nine months. They are trained in the methodology, during a two-day workshop, based on an experiential, i.e. embodied-learning approach.

### Participants

Table 1 shows an overview of the number of participants. For a total of 2183 children who participated in TeamUp sessions between September 2018 and July 2019, attendance was registered. A subgroup of these children were in sessions that had just started at the time of the study (n = 209 children). A total of 22 teams of facilitators in 15 asylum seekers centres were observed (n = 81 unique facilitators; 67.9% female, mean age 34.7 and range: 19–69 years). Of these, 29 facilitators were observed twice, leading to a total of 110 observations. At T1, 55 facilitators were observed, with 18 facilitators observed twice, leading to 73 observations in total. At T2, 62 facilitators were observed, with 21 facilitators observed twice, leading to 83 observations in total. Given the voluntary nature of the facilitator role and some turnover in facilitators, the majority of teams consisted of different members at each time point. A total of 73 trainees (71.2% female, mean age 30 and range: 19-68 years) participated. Trainings had an average of 14.6 participants. Almost all (n = 68 individuals; 93.2%) completed the pre and post training assessments. A total of 99 facilitators (80.8% female; mean age 35.9 and range: 19–72 years) responded to a survey, sent to 190 facilitators via email. The majority was providing sessions at the time of the survey (87.8%). Others had been trained in the TeamUp methodology, but had not yet facilitated sessions (7.1%) or had left (5.1%).

The focus group discussions (FGDs) and key informant interviews (KIIs) involved 6-14 asylum seekers centres/groups in which TeamUp had been implemented for an average of 12.6 months (range three to 26 months). We included; (a) 24 facilitators in six FGDs (87.5% females; aged 21–72; predominately of Dutch nationality); (b) 79 children in 19 FGDs (36.7% female; aged five to 17), with an average of 4.2 participants (range: 2-9 children) per goup; (c) 15 children and adolescents in 15 KIIs (20.0% female; aged seven to 15), due to specific native languages and children’s availability; and (d) 10 staff members in nine KIIs (30.0% females; aged 27-58; predominantly Dutch nationality), working for the ‘Centraal Orgaan Opvang Asielzoekers’ (COA) who manage the asylum seekers centres.

### Instruments

#### Attendance registration lists

Children’s attendance was registered in a log (attending/not attending), including name, gender and age.

#### Observation checklists

Observation checklists were developed to assess implementation quality, including; (a) facilitators’ individual-level fidelity with 20 items, on a 2-point scale (‘not done’ or ‘done’); (b) facilitators’ team-level fidelity with 16 items, on a 3-point scale (‘not done’, ‘partly done’ or ‘very well done’) to assess the degree to which the intervention was implemented as it was designed [7] and, (c) facilitators’ competencies with nine items, on a 4-point scale (‘harmful’, ‘absence of competency’, ‘competency partly present’ and ‘done with mastery’) assessing a set of common clinical competencies required when working with children (based on ongoing work by Kohrt et al. [35]; Kohrt et al. [34], Jordans et al. [32], Ottman et al.[48]). The combination of these tools aimed to evaluate implementation quality or service delivery as executed by the TeamUp facilitators. Prior to the study, the master trainers, who formed part of the intervention development team and conducted the observations during the study, completed and scored 10 observations which yielded a moderate inter-rater reliability (IRR) (Kappa = .52). In addition to the observer-rated facilitator fidelity, we used, (d) self-rated observation check-list for facilitators. These contained the same 36 items and asked them to score themselves and their team. All facilitators filled out the self-rated observation checklists after being observed.

#### Training evaluation

Training was evaluated using a pre-posttest assessing perceived self-efficacy for the implementation of TeamUp. This included 16 items, with a 5-point Likert-scale and additional options of ‘I don’t know’ and ‘I don’t want to say’. The questionnaire inquired about trainee’s perceived ability to interact with a group of multi-lingual/cultural children, work in a team, and implement specific session elements and handling children’s strong emotions and challenging behaviour.

#### Facilitator survey

We designed an online survey to assess and understand facilitators perceptions regarding their motivation and satisfaction, self-efficacy, perceived impact on children, experienced burden and perceived quality of offered support. This survey included 42 questions of which 25 were quantitative, on a 5-point Likert scale.

#### KIIs and FGDs

Topic guides for KIIs and FGDs were developed in English, and—for the use with children—translated into Arabic, Farsi, Tigrinya, Dutch and Armenian. All topic guides followed a similar format, including questions on positive and negative experiences with, or impressions of, TeamUp, possible reasons for (non-)attendance, as well as perceived impact of TeamUp. FGDs with facilitators also covered implementation challenges and perceived quality and availability of support. The KIIs with COA personnel also inquired about access barriers and challenges, and perceived added value to other activities offered at the asylum seeker centre.

### Procedure

#### Attendance

Attendance was taken by facilitators, registering children’s attendance at the start or during each session. Some COA staff provided basic personal information (age, gender) to support the registration process. Other facilitator teams based the lists on children self-reports.

#### Facilitator fidelity and competencies

Master trainers [KV, WV] conducted unannounced observation visits at two time points, with roughly two months between them. They observed facilitators individually and as a team throughout the sessions and rated them using developed checklists. For the 12 newly started groups, the second (or in one case the third) session was observed, for long-term running groups a random session was observed. Subsequent to each session observation—at T1 and T2—all facilitators were requested to complete their self-rated observation checklists, without discussing with their team members.

#### Trainee perceived self-efficacy

Prior and following initial two-day trainings the questionnaire was handed out for participants to complete.

#### Facilitator perceptions

An anonymous online survey, using Survey Monkey (https://www.surveymonkey.com/) was sent by email to all 190 facilitators who were registered as “active” or “on-hold” in an internal database at the time of circulation (October/November 2018). The survey was online for a six-week period, three reminders were sent.

#### Stakeholder perceptions

For the FGDs with the children, we selected, trained and guided seven research assistants, speaking five languages (Dutch, Arabic, Tigrinya, Farsi/Dari and Armenian). All research assistants spoke the native language of the children, had themselves lived in asylum seeker centres and most also had previous experience in conducting interviews in the centres. Hence, they were familiar with the setting, aware of possible power dynamics and the needed sensitivity with the children. Within each asylum seekers centre, we employed convenience sampling, depending on the availability of research assistants, their languages, children’s interest and feasibility factors. FGDs and KIIs with children were conducted in seven languages (additionally, French and English). The FGDs with facilitators were held in English and Dutch, the KIIs with COA personnel in Dutch only. All FGDs and KIIs were audio-recorded, transcribed and translated by the research assistants, other WCH staff or external volunteers.

### Analyses

#### Quantitative analyses

The quantitative data were analysed using MS Excel and Statistical Package for Social Sciences (SPSS) and consists mainly of descriptive statistics. Attendance indicators were calculated in two ways: (a) for all 42 groups as the average number of children in a session, and (b) for eight newly started groups as the percentage of sessions which children attended over a period of 12 weeks. Observed facilitator fidelity was analysed for each individual as the percentage done and not done on each session element during a session (individual-level), and for each team as the percentage very well done, partly done, and not done on each session element during a session (team-level). Also, at the individual-level, fidelity observations were compared to fidelity self-reports through correlations per session element. At the team level, the consistencies in facilitator responses within a team were analysed and compared to the observations at the team level. Facilitator competencies were analysed as the percentage of harmful behaviour, absence of the competency, partial presence of the competency, and full mastery on each of the competencies. A paired samples *t* test was conducted for the subsample of facilitators observed at both T1 and T2, split into less experienced (less than 4 months) and experienced (at least 4 months). The training evaluation was analysed per item (Table S1 in Additional file 1). Also, a paired-samples *t* test was conducted for the subsample with sufficient data pre- and post-training. Finally, the facilitator survey was analysed as the percentage of each response category on each item.

#### Qualitative analyses

Two authors (AB, KA) conducted framework analysis on FGD and KII transcripts. A broad coding framework was developed, containing ‘parent’-codes such as acceptability, feasibility and outcomes including various ‘child’-codes (deductive), using the Qualitative Data Analysis Software NVivo. Additional open and axial coding enabled detecting and linking emerging themes (inductive). Due to the large amount of data and variety of stakeholders interviewed, the second layer/round of coding, summarising and condensing was done on paper. The main findings and illustrative quotes were presented combined with the quantitative results (affirming or contradicting) to portray the mixed-method approach.

## Results

### Implementation process

#### Attendance

Attendance lists of 42 TeamUp groups over a period of six to 39 weeks included a total of *n *= 2183 children (*n*_*girls*_= 948, *n*_*boys*_= 1109, *n*_*missin*g_ = 126). More boys attended TeamUp sessions (50.8%) than girls (43.4%). A session was attended by a mean of 8.5 children (*M*_*girls*_= 4.2, *M*_*boys*_= 4.1), with large differences across centres and groups (ranging from 1 to 31 children per session). Table 2 shows the attendance percentages of eight newly started TeamUp groups, considering only children whose attendance was registered for the whole period of 12 weeks. Children attended on average 31.3% of the sessions during the 12 weeks period, translating to 1.4 times per month. Overall, more boys (52.2%) than girls (40.2%) were reached by TeamUp activities, while girls joined more frequently (38.4%) than boys (28.6%). The majority of the children (75.1%) attended one to four sessions within their first 12 weeks.

**Table 2:** Attendance percentages for newly started TeamUp sessions

	n	Percentage of sessions attended (12-week period)
Overall	209	31.3
Girls	84 (40.2%)	38.4
Boys	109 (52.2%)	28.6
Missing data	16 (7.7%)	12.0
Age groups^a^		
6–9 year-olds	62 (29.7%)	27.5
10–14 year-olds	78 (37.3%)	40.9
15–17 year-olds	22 (10.5%)	37.6
Missing data	47 (22.5%)	17.2

	n	Percentage of children attending
Number of sessions a child attended in 12-week period	209	
1–2 sessions		50.7
3–4 sessions		24.4
5–6 sessions		9.1
7 or more sessions		15.8

^a^Individual children’s age is self-reported or based on available registers, thereby 22.5% missing values

#### Reasons for non-attendance and suggestions for improving attendance

Most interviewed children reported their frequent participation in TeamUp sessions and stated diverse potential reasons for non-attendance, including official appointments, school and family commitment, other concurrently offered activities, being ill or having forgotten about the session’s starting time.

*“I didn’t even know that there was TeamUp today. I just happened to see a TeamUp facilitator in the hallway and they told me. We didn’t even know”,* another adds that *“I only found out because I came down stairs looking for my friend. And someone told me that there was TeamUp”* (nine to 11 year-old Farsi/Dutch-speaking girls).

COA personnel mentioned to suspect the lack of structure within the reception centre context, ongoing stressors for families and unawareness of the offered services at the centre, to possibly explain low attendance. Facilitators mentioned the challenges of mobilising children for the session, not having sufficient time prior to the sessions as well as the requirement of positivity, leadership and perseverance.

### Implementation quality

#### Facilitator fidelity: individual-level

Table 3 shows for each of the required session elements the percentage rated as “done” and “not done” following the session observations. Note that due to the different team compositions at each time point the changes between T1 and T2 should not be interpreted as changes over time, but as observations on two occasions. Overall, 49.2% of the observations showed the required session elements as ‘done’ at T1 and 58.2% of the observations showed the required session elements at T2.

**Table 3:** Percentage individual-level fidelity for each of the session elements

Session elements	T1 (n = 73 observations)		T2 (n = 83 observations)	
	Not done	Done	Not done	Done
Mobilisation	54.8	45.2	58.6	41.4
Prepare w/team	11.0	89.0	10.1	89.9
Prepare safe physical space	34.2	65.8	20.3	79.7
Interaction walk-in w/children	34.2	65.8	30.7	69.3
Greet children	60.3	39.7	41.3	58.7
Say goodbye to children	46.6	53.4	16.9	83.1
Give positive feedback	64.4	35.6	49.4	50.6
Give opportunities to participate	19.2	80.8	20.5	79.5
Group collaboration	57.5	42.5	50.6	49.4
Support settling moments	83.6	16.4	92.8	7.2
Manage high energy	75.3	24.7	66.3	33.7
Indicate boundaries of play area^+^	74.5	25.5	57.1	42.9
Set rules and limits for play	61.4	38.6	63.3	36.7
Support excluded children^+^	36.5	63.5	38.5	61.5
Include children w/specific needs^+^	70.0	30.0	0.0	100.0
Address challenging behaviour^+^	39.0	61.0	33.3	66.7
Actively discuss and reflect	17.1	82.9	31.3	68.7
Discuss alarming behaviour^+^	64.7	35.3	61.9	38.1
Make referral to COA^+^	100.0	0.0	81.5	18.5
Follow child safeguard policy	11.4	88.6	12.0	88.0
Mean	50.8	49.2	41.8	58.2

Percentages are adjusted for missing values and NA, the items with > 30% missing data and NA at T1 and T2 are indicated with^+^

The three intervention elements most often observed as ‘done’ at both T1 and T2 were ‘preparing with the team’ (89.0 and 89.9%), ‘following the Child Safeguarding Policy’ (88.6 and 88.0%), and ‘giving each child an equal opportunity’ to participate during the session (80.8 and 79.5%). The three intervention elements least observed at both T1 and T2 were ‘providing settling moments’ (16.4 and 7.2%), ‘managing children’s high energy’ (24.7 and 33.7%), and ‘setting rules and limits for play’ (38.6 and 36.7%). Some of the session elements had a high number of not applicable (NA) and missing values (see Table 3) and should be interpreted with caution. The percentages were calculated by excluding the missing and NA scores, however, some of the session elements, like discussing alarming behaviour and making referrals, were probably scored as “not done” instead of NA in many observations, influencing the percentages.

All of the observed facilitators (n = 81) also completed self-report fidelity checklists. For the individual-level fidelity at T1, the correlations between the observed and self-rated items ranged between Spearman’s *ρ *= −.15 and *ρ *= .54. The highest correlations were for the items “including children with specific needs” (.54) and “discussing alarming behaviour” (.49), which were both scored as “not done” by many facilitators, likely inflating the correlations. The item “mobilisation” also had a relatively high correlation between the observed and self-rated scores, *ρ *= .42. The remainder of the correlations were around *ρ *= .2. At T2 the correlations showed a similar pattern but were somewhat smaller, with most of the correlations around *ρ *= .15. The disagreements in all items were because the self-rating was scored as “yes, we did this” while the observer scored “no, this was not done”.

#### Facilitator fidelity: team-level

Table 4 shows for each of the required session elements the percentages of the teams scoring ‘not done’, ‘partly done’ and ‘very well done’ during the sessions following observations. The observed teams did not necessarily consist of the same facilitators at each time point. Overall, the percentage of teams that showed ‘partly done’ and ‘very well done’ at T1 and T2 was 72.5% and 73.0%, respectively. The three intervention elements most observed in the teams were correctly using play materials (100.0 and 100.0%), the provision of middle/main activities (100.0 and 95.5%), and implementing sports-based and active activities (90.0 and 100.0%). The three least observed intervention elements were ‘offering creative movement and dance activities’ (22.7 and 31.8%), ‘offering body-awareness activities’ (36.4 and 27.3%), and ‘working explicitly on a specific psychosocial support theme’ (27.3 and 40.9%).

**Table 4:** Team-level fidelity for each of the session elements

Session elements	T1 (n = 22 teams)^*^			T2 (n = 22 teams)^*^		
	Not done	Partly done	Very well done	Not done	Partly done	Very well done
Opening: check-in	18.2	54.5	27.3	36.4	36.4	27.3
Opening: body warm-up	22.7	40.9	36.4	22.7	18.2	59.1
Middle/main act.	0.0	50.0	50.0	4.5	27.3	68.2
Closing: cooling-down	40.9	40.9	18.2	36.4	22.7	40.9
Closing: check-out	22.7	54.5	22.7	31.8	36.4	31.8
Sportive and active activity.	9.1	36.4	54.5	0.0	27.3	72.7
Creative and dance activity.	72.7	27.3	0.0	72.7	22.7	4.5
Body awareness act.	63.6	27.3	9.1	72.7	18.2	9.1
Correct use of materials	0.0	45.5	54.5	0.0	31.8	68.2
Use routine	22.7	50.0	27.3	27.3	40.9	31.8
Demonstration	27.3	59.1	13.6	22.7	54.5	22.7
Group organisation	9.1	36.4	54.5	4.5	27.3	68.2
Build-up of act.	27.3	50.0	22.7	13.6	54.5	31.8
Session flow	9.1	63.6	27.3	18.2	45.5	36.4
Adapt to age, needs etc.	22.7	36.4	40.9	9.1	50.0	40.9
Work on focus theme	72.7	27.3	0.0	59.1	31.8	9.1
Mean	27.6	43.8	28.7	27.0	34.1	38.9

^*^ Teams consist of different team members at T1 and T2

All of the observed facilitators (n = 81) also completed self-report fidelity checklists for their team (2 to 6 facilitators per team). Unfortunately, the inconsistencies between facilitators of the same team were so large that the results of the self-report per team could not be interpreted well and are therefore not reported.

#### Facilitator Competencies

Table 5 shows the percentages of the observations scored as ‘harmful’, ‘absence of the competency’, ‘competency partly present’ and ‘mastery’ at both time points. Overall, facilitators demonstrated higher ‘adequacy’ (defined as the sum of ‘partly’ and ‘mastery’) than ‘inadequacy’ scores (defined as the sum of ‘harmful’ and ‘absence of competency’). On average, 82.9% of the observations demonstrated adequate competency at T1 and 88.4% at T2. Particular strengths were observed on empathy (98.6 and 91.6% adequacy at T1 and T2, respectively) and team collaboration (98.6 and 97.5% adequacy at T1 and T2, respectively). The main observed weaknesses were the behaviour management of children (36.4% and 10.9% inadequacy at T1 and T2, respectively) and giving feedback (27.8% and 18.1% inadequacy at T1 and T2, respectively).

**Table 5:** Percentages of observations showing each level of competency

Competencies	T1 (n = 73 observations)				T2 (n = 83 observations)			
	harmful	absent	partly	mastery	harmful	absent	partly	mastery
Empathy	0.0	1.4	55.5	43.1	0.0	8.4	45.8	45.8
Connection	0.0	8.3	68.1	23.6	0.0	12.0	53.0	35.0
Non-verbal	0.0	12.5	62.5	25.0	0.0	15.7	53.0	31.3
Adaptable^+^	0.0	23.6	63.9	12.5	0.0	13.4	63.4	23.2
Feedback	1.4	26.4	61.1	11.1	0.0	18.1	59.0	22.9
Inclusive	0.0	23.6	55.6	20.8	0.0	6.0	60.3	33.7
Behaviour management^+^	0.0	36.4	56.1	7.5	1.2	9.7	61.4	27.7
Group management^+^	0.0	18.1	63.8	18.1	0.0	17.7	44.3	38.0
Team collaboration	0.0	1.4	40.3	58.3	0.0	2.5	37.3	60.2
Mean	0.2	16.9	58.5	24.4	0.1	11.5	53.1	35.3

Percentages are adjusted for missing values in the items indicated with a ^+^

When comparing facilitators who did a session at both T1 and T2 based on their experience, the competencies of facilitators significantly improved. Those with less than 4 months of experience, *t* [24] = 5.66, *p *< .001, (*M*_*difference*_ = 3.08, 95% CI = 1.96 to 4.2), showed a large effect (Cohen’s *d *= 1.2) and those with at least 4 months of experience, *t* [20] = 1.76, *p *= .043), improved with a small-medium effect (*d *= 0.48). Given the small sample size, some facilitators having been observed twice and their varying levels of experience as facilitator (1 month to 5 years), these results need to be interpreted carefully.

#### Self-efficacy pre-post training

On average, more respondents rated their ability to handle specific aspects of the TeamUp intervention as high or very high after the training (79.5%), compared to before (62.1%). The paired-samples* t* test showed that the total score increased significantly from pre- to post-training (*M*_*pre*_ = 44.5, *SD *= 6.7 and *M*_*post*_ = 49.4, *SD *= 6.7, *t* [39] = 5.42, *p *< .001). On most elements, participants rated their perceived self-efficacy higher after the training compared to before. Participants continued to perceive a few elements as challenging, such as dealing with children’s strong emotions and challenging behaviours, as well as implementing movement-based and creative activities, and energy-release activities (see Table S1 in Additional file 1 for more details).

### Perceptions on implementation and outcomes

#### Facilitator perceptions

Based on the reporting of the surveys amongst the facilitators (N = 99), we learned that they enjoyed their role (84.8%), the interaction with the children (89.9%) and their team (75.8%), see Table 6. In line with the observed fidelity items, survey respondents felt more comfortable providing sports and active games (85.9%), compared to (creative) body-movement activities (59.6%). Many facilitators noted feeling uncomfortable with being the centre of attention, a few were positive about gaining confidence over time. A third to one-fourth of respondents stated to experience a little difficulty or stress (27.5–41.3%) in their role, fewer reporting a lot and very much difficulty or stress (3.3–11.6%). Particularly, dealing with the children’s behaviour, hearing children’s experiences, noticing emotions or behaviours were regarded as difficult or stressful.

**Table 6:** Perceptions of facilitators in percentages (n = 99)

Facilitator perceptions	Not at all	A little	Neutral	A lot	Very much
Motivational and satisfaction factors					
Liking facilitator role	0.0	2.2	5.5	37.4	54.9
Liking interaction with children	0.0	0.0	1.1	28.9	70.0
Liking working in their team	1.1	2.2	14.3	30.8	51.6
Feeling appreciated and recognized by TeamUp	1.1	6.8	21.6	31.8	38.6
TeamUp to benefit them personally	2.3	3.4	12.6	48.3	33.3
TeamUp to benefit them professionally	5.7	8.0	22.7	43.2	20.5
Self-efficacy					
Comfortable with sports/games	0.0	2.2	4.4	36.3	57.1
Comfortable with body-movement	1.1	8.7	26.1	40.2	23.9
Comfortable with body-awareness	0.0	3.3	18.5	43.5	34.8
Comfortable with role/responsibilities	0.0	2.2	7.7	46.2	44.0
Burden (difficulty or stress experienced)					
With children’s stories/experiences	19.8	37.2	31.4	10.5	1.2
With children’s behaviours	19.6	41.4	28.3	10.9	0.0
With running session	38.5	38.5	19.8	3.3	0.0
With team members	49.5	27.5	17.6	1.1	0.0
With remembering TeamUp session/content	34.1	40.7	17.6	7.7	0.0
Perceived support					
Usefulness of team/intervision meetings	0.0	3.4	13.8	39.1	43.7
Support from Volunteer Coordinators	0.0	3.4	12.6	29.9	54.0
Support from Senior Trainers	0.0	4.6	13.8	35.6	46.0
Usefulness of information/communication from TeamUp team	2.3	4.6	28.7	42.5	21.8

Perceived impact	Very negative	Negative	Neutral	Positive	Very positive
On children’s emotional wellbeing	0.0	0.0	5.6	67.8	26.7
On children’s behaviours	0.0	2.2	8.9	75.6	13.3
On children’s social abilities or relations	1.1	0.0	6.7	63.3	28.9
On children’s emotional regulation	0.0	0.0	24.7	70.6	4.7
Children feeling at ease	0.0	2.2	18.7	61.5	17.6

Percentages are adjusted for missing data and “don’t know/don’t want to say” replies, max percentages of these is 14.1%

While managing children’s behaviours and being confronted by children’s experiences was difficult, this in turn also motivated facilitators to continue with TeamUp. Noticing children’s enjoyment during the sessions appeared to be a primary motivator. A few volunteers stated to perceive a strong benefit of TeamUp for the participating children – TeamUp *“transforms difficulties into positivity for the things we do for them.”* and another expressed that *“through these activities we help [the children] to forget for a little [ongoing worries] while making contact and playing with the child.”* Overall, facilitators perceived TeamUp to have a positive to very positive impact on children’s emotional wellbeing (85.9%), behaviours (80.8%) and social abilities or relations with other children (83.8%). One survey participant stated *“we [facilitators] are making a difference in their [the kids’] lives, however small it may be”*. Moreover, participating facilitators were positive about the TeamUp intervention, its methodology, capacity-building and mentoring support structures.

#### Evaluation of facilitators

Most children spoke very positively about the facilitators, describing them as ‘nice’, ‘good’ or having ‘passion’. Some mentioned facilitators’ irritability, lack of fairness, inconsistency or leniency with the enforcement of session rules and discipline. COA personnel strongly appreciated the enthusiasm, dedication and self-reliance of facilitators. A few staff desired more depth in the intervention, training, or increased facilitator self-confidence, calm or a more suitable dress code for facilitating movement-based activities. All stakeholders were critical of facilitator turnover, indicating its effect on quality and therefore impacting children’s sense of trust, relationship-building between children and facilitators as well as within facilitator teams and the collaboration with COA.

*“they [children] need a safe environment. Once this is not safe, they will show other behaviour, do other things, they will walk away […] If they [children] come back to tell their story, [it is] important that there is trust. […] then part of the team are leaving again. This happens… In general a lot collapses and you have to rebuild this. […] It costs a lot of time and energy.”* (COA staff, centre in Gilze)

Respondents from all stakeholder groups, expressed the added value of having at least one man and/or someone with a migrant background within the facilitator team, allowing children to have a diversity of role models. Trust, bonding and showing genuine interest and care was said to be vital to the facilitator role and overall methodology. COA personnel expressed valuing facilitators’ independent and neutral role, offering children attention, interaction and connection with adults who are external to the asylum seeker centre.

#### Perceived implementation challenges

Managing children’s diverse levels of energy and children displaying strong emotions or challenging behaviour during TeamUp sessions appeared to be one of the primary difficulties for facilitators. The open group nature of the intervention results in varying group sizes and composition of children participating in each session, requiring facilitators’ continuous flexibility and adaptation to children’s individual and collective needs.

*“[When] we feel the energy [is getting] really high, [then we are] sitting down, smell the flowers [breathing exercise where children are guided to pretend to smell a flower, thus breathing in deeply]. Sometimes they are just hyper*-*energetic. And then we are like ‘oh we need something to stop this’. First we sit there and they roll over the groups [of children] (laughing), then after the ritual [e.g. clapping routine] they chill, they are quiet, so you can explain the next game or do something, really”* (facilitators, centre in Oisterwijk)

Other challenges included managing children’s expectations, their diverse requests for different activities, finding suitable activities to engage teenagers and to prevent below 6 year-olds from joining the sessions (given the target age range of the TeamUp intervention). Survey respondents also mentioned language barriers and request for e.g. native Arabic or Farsi-speakers within the team. In line with the observations, facilitators explained that session implementation often depended on team stability, dynamics, communication and collaboration. In turn, high turnover of facilitators usually impeded this team work. Usually, session implementation quality and rapport between facilitator and children improved over time (also see section on competencies). Despite the experienced difficulties, most facilitators—who participated in the survey and/or FGDs—voiced appreciating and growing within their role. They perceived challenges and learnings as rewarding and motivating.

#### Suggestions for implementation improvements

Children’s feedback differed and provided concrete ideas for improvements. Generally, children desired sessions to be longer and more frequent. Many also requested fewer activities, each lasting for a longer time. In their opinion, changing activities frequently within a session seemed to break the momentum and resulted in frustration or reduced satisfaction. Several respondents suggested the repetition of activities to offer children predictability and structure. A few children felt that explanations and instructions of activities took too long, despite TeamUp’s facilitation methodology of using non-verbal demonstration and flow to present new games and rules. Children alluded to their wish to be fully immersed in the activities, to feel a sense of achievement and recognition for their efforts. All stakeholders indicated a need for TeamUp to be adapted to meaningfully engage adolescents.

#### Perceived outcomes

All stakeholders perceived the TeamUp intervention positively. Activities allowed children to experience positivity and normalcy, release high energy or strong emotions (e.g. expressing anger), and build peer-relationships in a socially and emotionally safe space with trustworthy adults. Children would strengthen social-emotional abilities through *“a playful way of learning”*. COA staff particularly appreciated that TeamUp offered an additional referral platform, supporting their (social) work in the centres.

Children’s accounts of TeamUp sessions, alluded to them experiencing a time and space of (emotional) safety and normalisation, an opportunity to play and move, be seen, heard and taken into account, as well as to interact and connect with peers.

*“I like these games, it reminds me [of] when I was little”; “I feel like I am playing happily […] it reminds me of my country”* (7-10 year-old Arabic-speaking children). *“You can’t meet people if you spend your time at home, so coming here and meeting up with friends gives you a good feeling”; “it’s fun and it’s good for your health”* (15-17 year-old Tigrinya-speaking youth).

As children often described the activities in great detail, requiring focus and coordination, alludes to them experiencing a sense of being in the present moment. This might also indicate that children were fostering their social, cognitive and physical abilities (e.g. movement and playing resources) during TeamUp sessions. Several interviewed children expressed their need for more fairness and consistent rules.

*“I used to fight when I was at the former [*asylum seeker centre*] but I don’t fight anymore”*; *“[when someone annoys me], I feel like I don’t want to play with them”*; “*I don’t fight [when someone else pushes me], I just tell him to leave me alone”*. (6-17 year-old Tigrinya-speaking children).

Facilitators described various individual children showing improved self-regulation and behaviour over a period of time and when attending sessions regularly. For example, children showed a reduction of displayed irritability, anger, aggression or frustration when losing a game. Some also increasingly listened to the instructions, were able to choose to take a “time out” or to apologise to others after conflict. Other perceived changes in children were increased participation, interaction, collaboration, and trust, e.g. holding hands or playing with peers of another gender and/or ethic group. Facilitators and COA staff observed children exhibiting more self-confidence, and appearing more comfortable and relaxed within the session environment, usually after weeks of shyness and reluctance to join in. A few children seemed to increasingly “*feel freer to be themselves*” or getting out of their “*comfort zones*”. Hence, they explored boundaries with facilitators, or expressed their needs, e.g. suggesting new or adaptation of activities. This was perceived as an increase in sense of assertiveness and agency.

*“Once it just happened that there was just girls [in the session] and they asked ‘can we dance, but can you close the blinds? So people can’t look!?’ and […] we didn’t even think about that* - *that [this] would [could] have been a barrier. So it’s just one of those things, as you go along. And there was a point where the girls would say ‘now I want to teach everyone this move’ and they were leading [them]”* (facilitators, implementing sessions in various centres)

Despite giving various examples, facilitators were reluctant to make general statements about improvements observed in children’s behaviour. For example, children continued to manifest impulsive, aggressive and bullying behaviours throughout the sessions. Even the interviewed children expressed their frustration and disappointment about the aggressive behavior of peers, whilst also describing their own readiness to respond physically when feeling angry, upset or annoyed. Some conflicts were challenging to mitigate, especially due to communication (e.g. language barriers), the attendance of different children every week and frequently rotating facilitators. In spite of these challenges, all facilitators perceived TeamUp to support and contribute to children’s socio-emotional learning, peer interaction and psychosocial wellbeing. While COA staff were usually not present during TeamUp sessions, they argued that TeamUp positively contributed to children’s socio-emotional development and psychosocial wellbeing.

## Discussion

The current study showed mostly positive results for the intervention’s quality of implementation. Facilitators exhibited moderate to adequate fidelity and high competencies. Stakeholders perceived the outcomes and implementation as overall positive, suggesting TeamUp to contribute to children’s psychosocial wellbeing as well as providing insight for intervention fine-tuning. Attendance and group size were low on average, alluding to a need for increased mobilisation or fixed, closed groups.

First, the implementation process demonstrated that children attended on average one-third of the sessions and 8.5 children joined per session. The majority of the children attended one to four sessions within their first 12 weeks. These results showed lower attendance than anticipated. On the one hand this may show that children perceived the open group nature of the intervention as a choice-making opportunity [47]. More so, this might also indicate the high mobility and frequent relocation of refugee families within the Netherlands. Still, these findings call for improved monitoring, mobilisation and intervention adaptation, as regular attendance and active engagement are likely to impact positive outcomes [56, 60]. Children also mentioned forgetting about the start time of TeamUp session. This appears reasonable given ongoing daily stressors for families and limited structure and predictability within the asylum seeker centre setting [18, 37]. One solution may be to implement TeamUp to existing and fixed groups of children. More attention could also be given to ‘mobilisation’ to promote the session start. Lack of awareness rather than interest may explain low attendance. Engaging children as ‘peer mobilisers’ to encourage others to join TeamUp as well as more visibility or parental involvement were proposed by the interviewed stakeholders to promote the intervention and increase children’s attendance. Nevertheless, feedback from adolescents may suggest the need for adaptations to strengthen participation and meaningfully engage this specific age group in the sessions.

Second, the evaluation of the ‘quality of implementation’ dimension rendered mostly positive results and a number of learnings. Facilitator fidelity was moderate to adequate with individual-level fidelity rated as ‘done’ for an average of roughly 50% and team-level fidelity rated as ‘partly done’ and ‘well done’ for an average of roughly 70%. Although an a priori threshold was not set, a minimum of 80% was regarded as a desired level of fidelity by the research team based on similar studies [34, 35]. The difference between individual-level and team-level fidelity may have various explanations: The high inconsistencies within teams and between observer-rated and self-rated fidelity made it difficult to draw conclusions about facilitators’ perceived fidelity. Facilitators might have needed prior training on the fidelity tool’s self-rating. Also, as TeamUp is meant to be delivered by a team of facilitators, implementing as well as rating the sessions on a team-level might have been more appropriate. Further, the team-level items might have had a stronger focus during the facilitator trainings compared to the individual-level items.

Overall, the session structure (e.g. opening, middle and closure) and assuring main activities, were implemented well by all teams. However, the integration of settling moments (e.g. routines, cooling-downs) during the session, the management of children’s energy level (e.g. moving from high energy to low energy) and setting boundaries (e.g. rules and limits for play) proved to be most challenging for facilitators. Facilitators’ team-level fidelity demonstrated limited implementation of elements such as creative movement, body-awareness exercises, which are core to the neurophysiological and body-based approach of TeamUp [36, 52, 61, 69], as well as the explicit utilization of the eight psychosocial themes. Through the survey we found that facilitators experienced limited comfort, confidence and “know-how” when delivering these types of activities, and trainees showed lowest perceived self-efficacy on these elements, even post training. These are key elements to the intervention (cf. [16] to enhance a safe physical and emotional space in order to promote mind–body connection and healthy relationship with oneself and others [27, 38, 51, 69]. Hence, these deserve further attention in future capacity-building and mentoring support for TeamUp facilitators.

Facilitators’ competencies were high, averaging around 85% adequacy. Facilitators depicted strong competencies in empathy and team collaboration. As competencies were assessed during session implementation, this suggests strong recruitment and selection of suitable facilitators as well as possibly supportive competency mentoring ‘on the job’. The competency least demonstrated was behaviour management of children. This may indicate that this is a skill obtained ‘on the job’, while implementing sessions and learning how to manage the behaviour of the children throughout the session in collaboration with the other team members. Trainee’s perceived self-efficacy improved significantly following training, facilitator satisfaction and self-efficacy were high, experienced burden was low, and perceived capacity-building support was high. This demonstrates the TeamUp intervention to offer strong and useful trainings and mentoring for facilitators.

Third, all stakeholder were positive about the TeamUp intervention and its potential impact on children’s socio-emotional learning, peer interaction and psychosocial wellbeing. The data provided insights on the need to deepen the intervention’s development, methodology and implementation (i.e. use of themes, demonstration, energy modulation). Children’s feedback varied widely on preferred activities and suggested improvements for TeamUp. Diversity and group heterogeneity might have contributed to this. Children’s honest expressions portrayed their agency and trust in sharing their TeamUp experiences with the research assistants. All stakeholders gave specific examples of how TeamUp fostered children’s sense of normalcy, safety, socio-emotional learning and psychosocial wellbeing. COA personnel particularly appreciated the intervention’s psychosocial framework and its supportive referral platform. However, given the frequent change in groups and reported aggression and conflict during the sessions, this may indicate children’s ongoing difficulties to self-regulate, and the need for more sessions and predictability in group composition for increased impact. Children’s low and irregular attendance and the open group structure of the intervention might have affected implementation quality and likely potential outcomes [5, 56]. Whether regular or increased attendance would lead to quantifiable outcomes, will require further research.

There were several limitations to our study. Primarily, we used real-time structured observations to assess facilitator’s intervention fidelity and competencies [13, 14, 32, 34, 35]. While observations hold various known biases, the method was practical and realistic for the current study. Furthermore, our observations tools showed merely moderate IRR (Kappa = 0.52) prior to data collection and we proceeded due to timing and logistical reasons. Some items and distinction between elements remained unclear for observers, possibly contributing to the noted differences at T1 and T2. The “not applicable” answer option for some items, might have caused confusion. Moreover, it proved challenging to observe and rate 2 to 6 facilitators simultaneously, particularly within the 1-hour session time frame. The study demonstrated substantial turnover of facilitators, with less than half of them being observed at two time points. In future studies, observing only one facilitator and fixed teams at both times, would be more reliable. The turnover of children and facilitators did not only challenge data collection and analyses, but more so affected group development stages for children and facilitator teams [24, 68] and in turn likely affected implementation quality and potential impact (i.e. safety, predictability, trust, rapport, group cohesion and sense of connectedness). Increased efforts were made during recruitment and training of facilitators by emphasising the need for a minimum of nine months of commitment to the role. As a result of the study, facilitator turnover is more closely monitored and discussed bi-annually. In addition, regular facilitator discussion days and trainings are offered to strengthen facilitators’ competences, confidence, opportunities for growth and commitment. Since session facilitation is offered by a team, the continuation of activities may at times take priority over the stability of individual facilitators. In other countries where TeamUp is implemented, facilitators receive small stipends and/or TeamUp activities are integrated into their existing role (e.g. teachers, social workers) in order to address this issue. These challenges will be addressed in subsequent studies and learnings applied for future implementation of the intervention. In addition, we could have applied a more comprehensive evaluation framework such as Moore et al. [46]. Since our study did not assess outcomes quantitatively, causal mechanisms and contextual factors associated with the variation in outcomes will be explored during or after the examination of effectiveness.

Despite the limitations and challenges, the study unpacked and evaluated the complex and interrelated processes of TeamUp sessions and provides inspiration and learnings on the added value of integrating the body and non-verbal modalities, such as movement-based psychosocial interventions, globally. Monitoring systems need to be relevant and user-friendly to generate valuable data in order to develop, promote and assure quality interventions [26].

## Conclusion

A process evaluation was conducted to examine the implementation process, implementation quality and perceived outcomes of TeamUp, a movement-based psychosocial intervention for refugee children, in the Netherlands. Our work strongly links research and practice with the aim to contribute to increased quality and accountability of interventions in the humanitarian sector. The main conclusions from this study are: (1) The implementation process exhibited lower average attendance and group size than expected, demonstrating a need for increased mobilisation and encouraging closed and stable groups (2) The intervention’s implementation quality showed facilitator fidelity ranging from moderate to adequate and competencies being high—indicating strong selection and need for specific fidelity strengthening. Trainee’s perceived self-efficacy improved significantly following a two-day training and facilitators showed high satisfaction and self-efficacy, low experienced burden, and high perceived capacity-building support. Gaps will be addressed by the intervention team with specific capacity-strengthening and facilitator retention with ongoing and future implementation. (3) The TeamUp intervention was regarded positively by all stakeholders overall, perceiving a positive impact on children’s socio-emotional learning, peer interaction and psychosocial wellbeing. Future research and evaluations will need to balance rigorousness and pragmatism, to inform content development, implementation and scaling of the intervention—to assure quality and impact. Further research is ongoing to examine outcomes quantitatively and assess the effectiveness of the TeamUp intervention. We thereby strive to contribute to increasing the evidence base of scalable psychosocial interventions for children affected by conflict and adversity.

## Supplementary Information

**Additional file 1: Table S1.** Trainee perceived self-efficacy pre and post training in percentages.

## Data Availability

The datasets analysed during the study are available from the corresponding author upon request.
