# Supplementary material for: Process evaluation of TeamUp: a movement-based psychosocial intervention for refugee children in the Netherlands
Source: Int J Ment Health Syst. 2021 Mar 19;15:25. doi: 10.1186/s13033-021-00450-6 (PMC7977563; doi:10.1186/s13033-021-00450-6)
Supplement: Supplementary file 1 — Additional file 1: Table S1. Trainee perceived self-efficacy pre and post training in percentages. [file 13033_2021_450_MOESM1_ESM.docx]

Supplementary Material

**Table A**

| *Trainee Perceived Self-Efficacy Pre and Post Training in Percentages* | | |
| --- | --- | --- |
|  | pre-training (n = 73 volunteers) | post-training (n = 73 volunteers) |

| Item | very low | low | average | high | very high | very low | low | average | high | very high |
| --- | --- | --- | --- | --- | --- | --- | --- | --- | --- | --- |
| Interact with children in reception centre | 0 | 1.6 | 32.3 | 51.6 | 14.5 | 0 | 1.6 | 15.6 | 56.2 | 26.6 |
| Communicate with non-Dutch speakers | 0 | 7.0 | 47.9 | 40.9 | 4.2 | 0 | 1.5 | 25.4 | 47.8 | 25.3 |
| Role and responsibilities | 0 | 0 | 22.7 | 65.2 | 12.1 | 0 | 0 | 7.8 | 60.9 | 31.3 |
| Plan session | 0 | 4.3 | 26.1 | 59.4 | 10.2 | 0 | 0 | 12.5 | 59.4 | 28.1 |
| Games/sports | 0 | 0 | 23.2 | 53.6 | 23.2 | 0 | 0 | 7.8 | 56.3 | 35.9 |
| Movement-based/dance activities | 0 | 12.7 | 42.3 | 36.6 | 8.4 | 0 | 3.0 | 19.7 | 51.5 | 25.8 |
| Tension/energy release activities | 0 | 5.8 | 40.6 | 40.6 | 13.0 | 0 | 0 | 22.2 | 47.6 | 30.2 |
| Cool down activities | 0 | 2.9 | 35.3 | 54.4 | 7.4 | 0 | 0 | 11.9 | 58.2 | 29.9 |
| Team work | 0 | 0 | 12.7 | 49.3 | 38 | 0 | 1.5 | 6.2 | 44.6 | 47.7 |
| Safe guarding | 0 | 0 | 25.8 | 48.4 | 25.8 | 0 | 0 | 6.1 | 40.9 | 53.0 |
| Limits of role | 0 | 4.6 | 38.5 | 43.1 | 13.8 | 0 | 0 | 13.4 | 44.8 | 41.8 |
| Personal limits | 0 | 2.7 | 23.3 | 53.5 | 20.5 | 0 | 0 | 17.9 | 55.2 | 26.9 |
| Deal with children’s emotions | 0 | 0 | 48.6 | 42.8 | 8.6 | 0 | 3.2 | 39.7 | 39.7 | 17.4 |
| Deal with children’s behaviour | 0 | 7.4 | 54.4 | 32.4 | 5.8 | 0 | 0 | 54.7 | 37.5 | 7.8 |
| Improvisation | 0 | 1.4 | 30 | 48.6 | 20 | 0 | 0 | 30.3 | 51.5 | 18.2 |
| Recognise symptoms of distress | 0 | 4.5 | 47.8 | 35.8 | 11.9 | 0 | 0 | 26.2 | 58.5 | 15.3 |
| overall % | *0* | *3.4* | *34.5* | *47.3* | *14.8* | *0* | *0.7* | *19.8* | *50.7* | *28.8* |

*Note: Percentages are adjusted for missing values and NA, max percentage of missing data / NA is 15%.*
